# Echocardiography in Cardiac Arrest: Incremental Diagnostic and Prognostic Role during Resuscitation Care

**DOI:** 10.3390/diagnostics14182107

**Published:** 2024-09-23

**Authors:** Alfredo Mauriello, Gemma Marrazzo, Gerardo Elia Del Vecchio, Antonia Ascrizzi, Anna Selvaggia Roma, Adriana Correra, Francesco Sabatella, Renato Gioia, Alfonso Desiderio, Vincenzo Russo, Antonello D’Andrea

**Affiliations:** 1 Cardiology Unit, Department of Medical and Translational Sciences, University of Campania “Luigi Vanvitelli”, Monaldi Hospital, 80131 Naples, Italy; alfredo.mauriello93@libero.it (A.M.); delvecchiogerardo@gmail.com (G.E.D.V.); antonia.ascrizzi@gmail.com (A.A.); annasroma08@gmail.com (A.S.R.); vincenzo.russo@unicampania.it (V.R.); 2 Cardiology and Intensive Care Unit, Department of Cardiology, Umberto I Hospital, 84014 Nocera Inferiore, Italy; gemmamarrazzo@gmail.com (G.M.); frasab93@gmail.com (F.S.); renatogioia@outlook.it (R.G.); alf.desiderio@tiscali.it (A.D.); 3 Intensive Cardiac Care Unit, San Giuseppe Moscati Hospital, ASL Caserta, 81031 Aversa, Italy; adrianacorrera@gmail.com

**Keywords:** cardiac arrest, echocardiogram, POCUS, echocardiography, post-cardiac arrest, ACLS, ROSC, CPR

## Abstract

Background: Cardiac arrest (CA) is a life-critical condition. Patients who survive after CA go into a defined post-cardiac arrest syndrome (PCAS). In this clinical context, the role of the echocardiogram in recent years has become increasingly important to assess the causes of arrest, the prognosis, and any direct and indirect complications dependent on cardiopulmonary resuscitation (CPR) maneu-vers. Methods: We have conduct a narrative revision of literature. Results: The aim of our review is to evaluate the increasingly important role of the transthoracic and transesophageal echocardiogram in the CA phase and especially post-arrest, analyzing the data already present in the literature. Conclusion: Transthoracic and transesophageal echocardiogram in the CA phase take on important diagnostic and prognostic role.

## 1. Introduction

**Figure 1:**
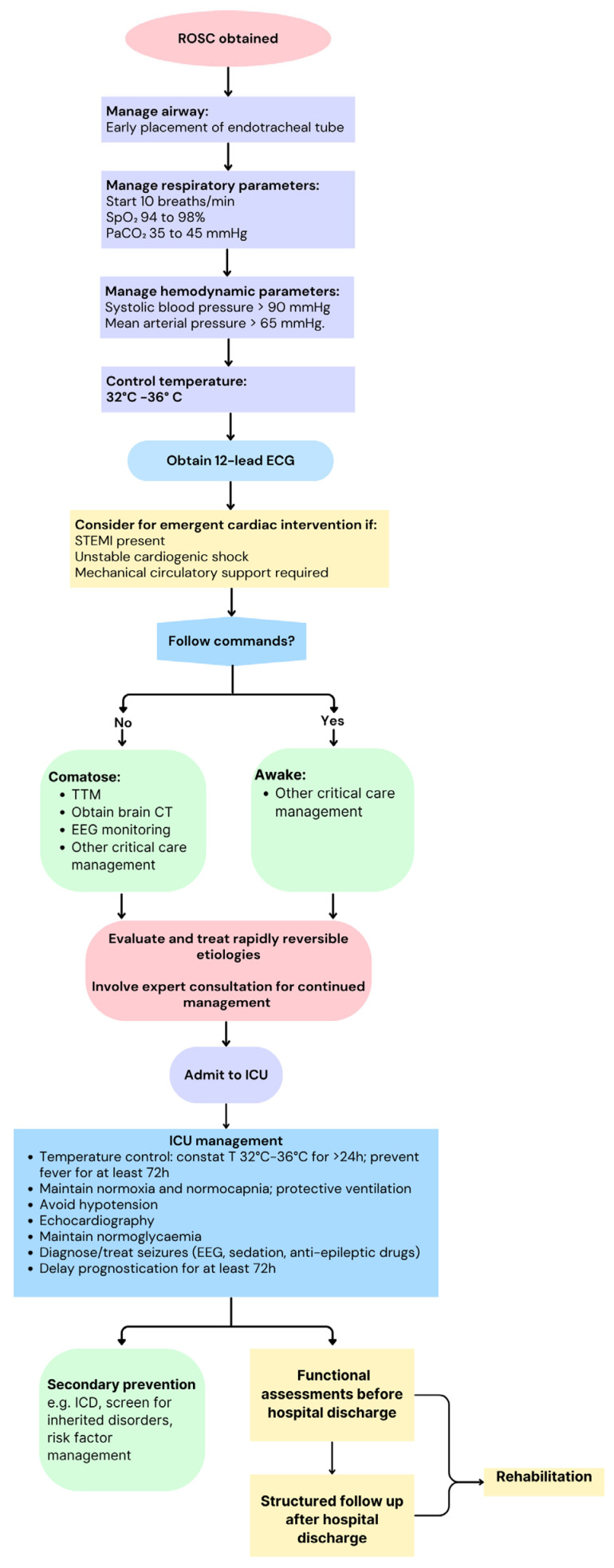
Adult post-arrest care algorithm. CT: Computed Tomography; ECG: Electrocardiogram; EEG: Electroencephalogram; PaCO_2_: Partial pressure of CO_2_; SpO_2_: Saturation of peripheral O_2_; ROSC: Return of spontaneous circulation; STEMI: ST elevation myocardial infarction.

Cardiac arrest (CA) is a critical medical emergency characterized by the sudden cessation of cardiac function, which results in the abrupt loss of effective blood circulation and brain and vital organ hypoperfusion, rapidly leading to death, if not promptly and effectively treated [1]. Despite advances in resuscitative efforts and post-arrest care, it has a significant morbidity and mortality. Both electrical and mechanical phenomena may be the primary cause of CA. Pulseless electrical activity (PEA) is a clinical state characterized by the presence of organized electrical activity on the electrocardiogram (ECG) in the absence of effective cardiac output (CO), resulting in no palpable pulse or measurable blood pressure [2]. This condition often arises from severe physiological derangements such as profound hypovolemia, hypoxia, acidosis, or cardiac tamponade [1,2]. The condition in which the palpable pulse is absent, but minimal cardiac mechanical activity is sufficient to generate some degree of CO is defined as pseudo-pulseless electrical activity (pseudo-PEA). Pseudo-PEA can be identified through advanced diagnostic modalities such as Doppler ultrasound or invasive hemodynamic monitoring [3]. Rapid and accurate assessment of cardiac function is pivotal for guiding therapeutic interventions and improving outcomes in patients experiencing CA. Echocardiography, particularly point-of-care focused echocardiography (POCUS), has emerged as an invaluable diagnostic and monitoring tool in the management of critically ill patients, including those in the peri-arrest and CA phases. The use of echocardiography in the clinical context of CA allows the real-time visualization of cardiac structures and function, aiding in the identification of reversible causes of CA, guiding resuscitative efforts, and facilitating informed prognostic decisions. The algorithm for adult post-arrest care is illustrated in Figure 1 [4].

Our review aims to summarize the current evidence about using echocardiography in the post-arrest setting.

## 2. Role of Ultrasonography in Cardiac Arrest

Ultrasounds (US) are essential in Advanced Cardiac Life Support (ACLS) protocols, both during CA and immediately following the return of spontaneous circulation (ROSC), as recommended by international guidelines [5,6,7].

In this specific setting, a “head-to-toe” POCUS strategy is recommended and includes echocardiography, transthoracic and transesophageal, and thoracic, abdominal, vascular, and brain US.

During cardiopulmonary resuscitation (CPR), a trained operator should perform US without impeding effective chest compressions and ventilation. The primary objective of the US during CPR is to optimize resuscitation efforts and guide the management by identifying potentially reversible causes of CA.

Following successful resuscitation, a more comprehensive US approach is warranted to optimize immediate hemodynamic functions, assist the ventilatory support, assess multiorgan complications, and investigate the underlying cause of the CA. Furthermore, it provides notable prognostic information [8].

Table 1 shows the main goals of the US during CPR and in post-resuscitation care.

### 2.1. Role of Ultrasonography during Cardiopulmonary Resuscitation

As already mentioned, during the critical phase of CPR, echocardiography can provide essential information, with a transthoracic approach being most used due to simpler equipment and greater accessibility, even for non-specialist physicians.

Several protocols exist for performing transthoracic echocardiography (TTE) during CPR, but a detailed description of these protocols is beyond the scope of this review and can be found elsewhere [9,10,11,12,13,14,15,16,17,18]. However, their core principles are identical, and no single protocol has proven superior. All protocols rely on using limited TTE windows to obtain simple yet crucial information without interfering with resuscitation maneuvers or prolonging CPR pauses [18].

The subxiphoid window offers the easiest visualization of the heart during chest compressions without interrupting them. The US operator could then take advantage of the pause during the pulse and rhythm evaluation for use in other views, keeping in mind that the pause should not exceed 10 s.

The US can evaluate the effectiveness of chest compressions by providing direct, real-time observation of cardiac chambers’ compression/relaxation. Adjusting hand placement for optimal chest compressions may be necessary, particularly if the area of maximal compression involves the aortic root or left ventricular outflow tract (LVOT), and the US can aid in optimizing the effectiveness of CPR [18].

Although the distinction between shockable [ventricular fibrillation (VF) and pulseless ventricular tachycardia (VT)] and non-shockable rhythms (asystole and PEA) is based upon ECG findings, in some cases the discrimination between asystole and fine VF is not obvious, because of a low signal amplitude. The detection of cardiac contractions by echocardiography can identify patients who may benefit from defibrillation, even if initially classified as “asystole” [19]. Moreover, the presence of detectable cardiac contractility distinguishes “true” electromechanical dissociation of PEA from “pseudo-PEA,” where in myocardial contractions are present but severely diminished, resulting in no detectable pulse or blood pressure. Although both conditions are categorized as “non-shockable” CA, their causes and prognoses may differ. Interestingly, regardless of the cause, pseudo-PEA cases have shown higher survival rates compared to those with no detectable cardiac motion, particularly in those with organized myocardial activity [20].

Therefore, the presence of detectable cardiac contractions could encourage physicians to continue the resuscitative effort and to consider an escalation in the advanced life support (like extracorporeal membrane oxygenation). Otherwise, the complete absence of cardiac motion, particularly if associated with large intracavitary thrombosis, could be an element in the decision to terminate CPR. Nonetheless, this decision must be made considering multiple factors beyond echocardiographic findings.

During resuscitation, identifying specific reversible causes can significantly impact survival, particularly in cases of non-shockable rhythms or refractory CA. US examination of the heart, lungs, abdomen, and proximal veins of the lower limbs can help detect common reversible causes such as pulmonary embolism (PE), cardiac tamponade, tension pneumothorax, and profound hypovolemia (Table 2) [18].

Given time constraints, US assessment must be highly focused and guided by clinical presentation. For instance, in trauma patients, the focus is on detecting tension pneumothorax and profound hypovolemia. In hypertensive patients with migrating chest pain, ruling out tamponade indicative of acute aortic syndrome is crucial. Similarly, in post-operative or oncology patients, priority is given to detecting massive PE [8].

Finally, US could be used to aid specific interventions during CPR, like insertion of peripheral or central venous cannulas, including those for extracorporeal life support (ECLS) or temporary pacing, drainage of cardiac tamponade, or confirmation of bilateral lungs aeration after endotracheal tube positioning [21,22].

Due to its retrocardiac location, transesophageal echocardiography (TEE) overcomes numerous difficulties encountered with TTE and is, therefore, a solid alternative. TEE provides continuous, high-quality imaging of the heart throughout resuscitation, expanding diagnostic capabilities by offering superior visualization of structures like the posterior pericardium for localized tamponade, the pulmonary artery for acute PE, and the thoracic aorta for aortic dissection. Additionally, TEE’s continuous imaging capability minimizes interruptions to chest compressions during pulse checks and rhythm analysis and the different positions of US operators interfere less with the resuscitative operations [23,24].

In a recent systematic review including 11 studies with 358 patients with both out-of-hospital CA (OHCA) and in-hospital CA (IHCA), Hussein et al. showed that TEE led to a diagnosis of the specifical reversible cause of CA in nearly one-half of cases (148 patients, 41%), with a diagnosis of PE as the most reported finding (43 patients) [25].

A focused TEE protocol and its clinical application in CA are shown in Table 3.

There are several limitations to the use of TEE during CA. TEE, compared to TTE, is an invasive technique with specific risks of oropharyngeal, esophageal, and gastric trauma related to probe insertion. Additional risks related to the use of CPR are unknown [25]. However, in this life-threatening scenario, the benefits probably overweight the risks. Moreover, the need for operators with great expertise and the specific equipment required limits the availability of this tool in the emergency setting. Finally, echocardiography should never interfere with resuscitation, or in this case, with ventilatory support. For this reason, TEE could be started only if the patient has already been intubated [26].

### 2.2. Role of Ultrasonography in Post-Resuscitation Care

The US, alongside other tools, such as ECG and point-of-care blood analysis, has a pivotal role and echocardiography should be performed in all patients as soon as possible, as recommended by international guidelines.

The main role played by echocardiography remains its utilization in identifying some of the most common CA etiologies, such as acute coronary syndrome (ACS), cardiac tamponade, cardiomyopathies, or PE.

Beyond the role in the identification of the underlying cause of CA, the US could aid airways and ventilatory support, as well as the optimization of hemodynamics. Moreover, it could point out possible complications of CPR, such as ribs or sternal fracture, pneumothorax, visceral trauma, bleeding, or iatrogenic aortic dissection [27]. Finally, cardiac and non-cardiac US have a role in prognostic evaluation.

#### 2.2.1. Diagnosis of the Underlying Cause of Cardiac Arrest

Echocardiography plays a pivotal role in diagnosing structural heart disease (SHD), both ischemic and non-ischemic, and PE, cardiac tamponade, and profound hypovolemia.

Arrhythmias caused by myocardial ischemia due to ACS are still the most frequent cause of CA [5]. Echocardiography has a major role in identifying ACS mainly by showing left ventricular systolic dysfunction with regional wall motion abnormalities (RWMA), particularly in the case of a non-diagnostic ECG.

During CA and CPR, detecting RWMA is only possible if myocardial contractility is preserved, but it is challenging even in these cases. Following ROSC, a fine evaluation of the regional myocardial contractility could be easier. However, RWMA in resuscitated patients is not a specific finding of ACS. Indeed, post-arrest myocardial dysfunction (PAMD), usually reversible, is common, even without coronary syndrome, and may occur with global wall motion abnormality or RWMA, including typical and atypical Tako-tsubo syndrome (TTS) patterns. Up to 60% of patients may experience PAMD, which typically resolves within 24–48 h [5]. Moreover, in the immediate post-ROSC, it could be difficult to differentiate new wall motion abnormalities due to ACS from pre-existing ones related to previous coronary events or cardiomyopathies. Finally, CA due to coronary artery disease (CAD) might occur without any RWMA, further complicating the scenario. Unsurprisingly, studies on echocardiography’s utility in diagnosing ACS/CAD after ROSC demonstrate variability in prevalence and diagnostic accuracy, highlighting a good sensitivity, but lack of specificity [28].

A retrospective, single-center, observational study at a tertiary hospital to evaluate POCUS performed by cardiologists within 60 min after was conducted by Elfwén et al. The study involved 617 adults admitted after a resuscitated CA. In this population, RWMA was detected in 37.6%, but only half of the patients who had undergone coronary angiography showed a culprit lesion [29].

More specific echocardiographic findings suggesting ACS as the cause of CA are the detection of mechanical complications of acute myocardial infarction (AMI).

Preferably, coronary interventions should be reserved for patients who do not have permanent severe neurological damage. Guidelines recommend immediate coronary angiography only in patients with evidence at ECG of ST elevation after ROSC. In other cases, coronary angiography should be considered after a multiparametric evaluation, encompassing patient history, clinical condition after ROSC, laboratory, and echocardiographic findings. None of them could be used alone, including echocardiography, keeping in mind that in the early phase of ROSC hemodynamic, ventilatory, and neurological support are priorities.

Acute PE might cause CA, mostly due to PEA or asystole, with an incidence of 2–7% in OHCA and 5–6% in IHCA [30]. Echocardiography has a major role in the diagnosis of PE, especially in patients with hemodynamic instability that could preclude the availability of pulmonary computed tomography (CT) [31]. The main echocardiographic features of PE are summarized in Table 4 [32].

**Figure 2:**
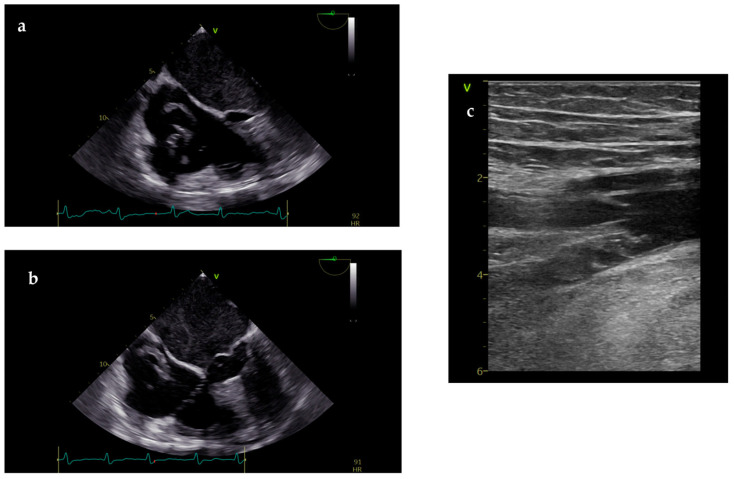
TEE performed during CPR in a patient with CA and non-shockable rhythm presentation, showing in-transit thrombus in right chambers suggesting PE as CA cause. Notably, the patient was affected by hypertrophic cardiomyopathy (**a**,**b**); vascular ultrasound performed in the same patient after cardiac resuscitation showed deep vein thrombosis involving superficial femoral vein (**c**).

However, right ventricle (RV) echocardiographic abnormalities frequently manifest following resuscitation from CA. Acute dilatation of the RV ensues within minutes from arrest, as blood shifts from the systemic circulation to the right side of the heart due to pressure gradients [29]. Beyond the role of echocardiography, vascular US of proximal veins of the lower limbs could be helpful in doubtful cases, with the identification of deep vein thrombosis (DVT), which increases the probability of PE as the cause of CA (Figure 2) [8]. Pregnant women are particularly at risk of cardiac arrest secondary to acute pulmonary embolism [33].

CA, mostly due to VT or VF, could be the first clinical manifestation of cardiomyopathies or myocarditis. Echocardiography in post-resuscitated patients could highlight typical features of these diseases. However, whilst the evidence of marked myocardial asymmetrical hypertrophy easily suggests the presence of hypertrophic cardiomyopathy (HCM), spotting typical features of other cardiomyopathies in the immediate post-resuscitative care is challenging due to the overlap with the biventricular systolic and diastolic abnormalities that characterize the PAMD [34,35].

Profound hypovolemia is among the most frequent causes of PEA, especially in traumatic CA. Moreover, in post-resuscitation care, fluid management is crucial for hemodynamic optimization. Echocardiography has a pivotal role in identifying characteristic signs of reduced intravascular volume, such as the evidence of small, hyperkinetic ventricles, with near end-systolic obliteration (“kissing ventricle”) along with a small or collapsed inferior vena cava (IVC), guiding fluid administration. Moreover, thoracic and abdominal US could be used to identify massive bleeding due to organ injury or aortic acute pathologies as the primary cause of CA or as a complication of prolonged CPR [20].

**Figure 3:**
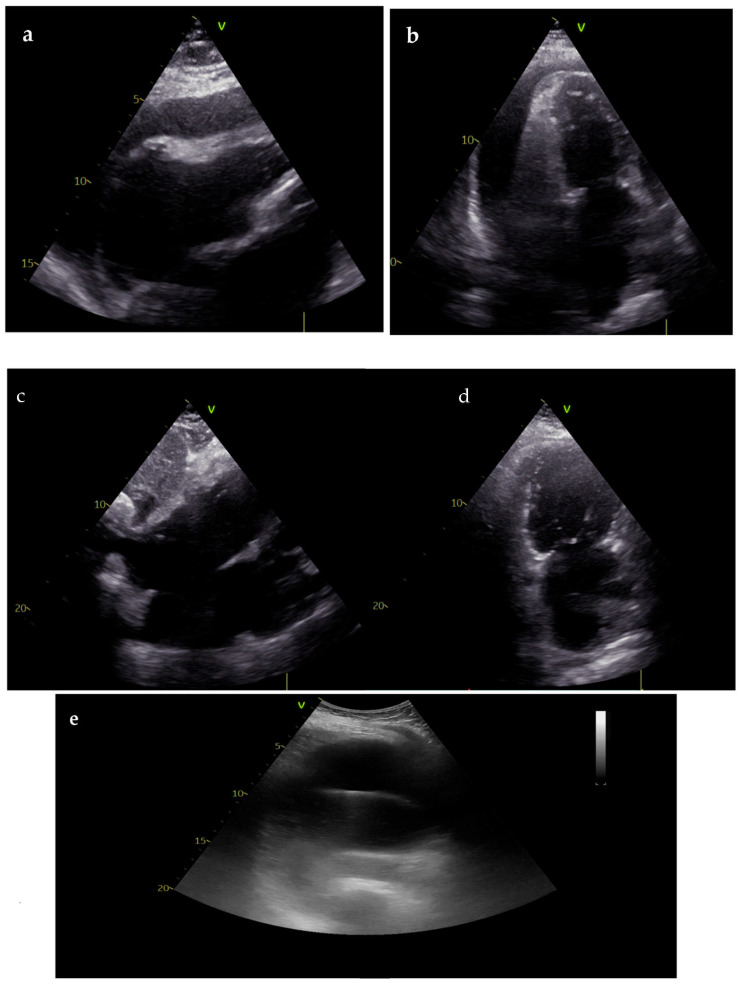
TTE performed during CPR showing cardiac tamponade as the cause of CA (**a**,**b**); after diagnosis, percutaneous pericardiocentesis with US guidance was performed leading to resolution of CA (**c**); TTE performed during post-resuscitation care showing complete resolution of pericardial effusion and tamponade (**d**,**e**).

Echocardiography is the first tool to identify cardiac tamponade, a common cause of CA with non-shockable rhythm. The diagnosis is usually performed during CPR with echocardiographic evidence of pericardial effusion, leading to cardiac chamber compression and eventually signs of ventricular interdependence. US is also used to guide pericardiocentesis. However, in the post-resuscitation phase, echocardiography enables the monitoring of successful drainage and heart decompression. Moreover, pericardial effusion and tamponade could represent a rare complication of CPR, which, if not identified, could compromise hemodynamic stabilization (Figure 3) [20,27,36].

However, clinicians should be aware that some relevant causes of CA do not have specific US findings, such as channelopathies or metabolic disorders. In these cases, echocardiography is still helpful to obtain important hemodynamic and prognostic information, but it is not diriment for diagnostic purposes.

Regarding hypothermia, an echocardiogram is used to confirm the diagnosis of CA caused by hypothermia. Hypothermia increases blood echogenicity, so a very slow flow of hyperechogenic blood in the heart cavities may mimic thrombus formations [37]. After ROSC, during normothermia, repetitive echocardiography is recommended to assess native cardiac output, left ventricular ejection, and aortic valve opening [37]. In addition, carotid bubble assessment after cannulation can be used to monitor body temperature during ROSC [37].

#### 2.2.2. Hemodynamic Monitoring and Optimization

After a successful ROSC, efforts are made to reach and maintain adequate central and peripheral perfusion and oxygenation. Pharmacological and mechanical cardiac supports (MCS) are often needed to achieve hemodynamic stabilization. Echocardiography provides a readily available and economical tool for bedside hemodynamic monitoring, guiding the use of fluids, inotropic drugs, vasopressors, vasodilators, and MCS and evaluating the impact of positive pressure ventilation. Systemic perfusion strictly depends on CO, determined by stroke volume (SV) and heart rate (HR). For its part, SV results from the interplay of preload, contractility, and afterload. Echocardiography is useful for obtaining information about all these factors and guiding therapeutic strategies (Table 4).

SV, and consequently CO, could be directly assessed by echocardiography using LVOT diameter and LVOT Velocity Time Integral (VTI). The LVOT-VTI can serve as a substitute for SV, thereby eliminating the measure of LVOT diameter as a source of error [38,39,40,41] Moreover, US enables the evaluation of fluid tolerance, identifying the grade of systemic and pulmonary venous congestion [using the venous excess US score and lung US (LUS)] [42,43]. The information on contractility is provided by the evaluation of left and right ventricular global systolic function, which can be performed using multiple parameters [43]. Finally, echocardiography might be used to estimate systemic and pulmonary vascular resistance, the main determinants of left and right ventricular afterload, showing a good correlation with invasive measurements [39,44]. Indeed, systemic vascular resistance (SVR) is determined using mean arterial pressure (MAP), right arterial pressure (RAP), and CO [44]. Similarly, pulmonary vascular resistance (PVR) could be calculated using pulmonary artery mean pressure (PAMP) and pulmonary capillary wedge pressure (PCWP) derived from the echocardiographic evaluation of the diastolic parameters and CO [45]. Furthermore, in recent years, there has been great interest in a non-invasive evaluation of right and left ventricular-arterial coupling, particularly in critically ill patients, such as post-ROSC [46,47]. Table 5 summarizes ultrasound parameters for hemodynamic monitoring and optimization.

### 2.3. Role of Transesophageal Echocardiography

TEE is indicated when TTE is unfeasible or inconclusive, especially in non-compliant ventilated patients in the intensive care unit (ICU) or those with a vascular cannula and thoracic or pericardial drainages limiting the echocardiographic views. Moreover, TEE should be preferred when it is not possible to mobilize the patient from the supine position. For this reason, there is a growing interest in utilizing TEE in intensive care settings, including in patients post-CA [23].

Even if performing TEE is not simple for beginners, a survey conducted by Arntfield et al. involving 14 emergency physicians without experience in TEE showed that, following brief, structured simulation-based training, they can proficiently obtain focused TEE images [48].

Similarly, a single-center retrospective observational study involving a total of 54 TEE exams performed by 12 emergency physicians between 2013 and 2015 revealed that TEE was feasible, safe, and clinically impactful. In this study, the most common indication for performing TEE was CA (43% during CPR and 26% in post-resuscitation care). TEE led to diagnostic conclusions in 78% of cases and impacted therapeutic decisions in 67% of cases. Notably, 55.6% of these examinations identified findings not easily visualized on TTE [49].

Another retrospective study, based on 274 TEE performed between 2012 and 2016 by 38 trained operators, demonstrated that point-of-care TEE in the ICU setting significantly influenced diagnosis and hemodynamic management decisions. Additionally, in nearly half of the patients who had undergone TTE within the preceding 24 h, the indication for TEE was inconclusive TTE, underscoring the technical limitations of TTE in this population [50].

A recent cross-sectional survey conducted by Teran et al. in the United States and Canada showed that the most common indication for performing TEE in the emergency department (ED) is assessing patients during resuscitation from CA (100%) and providing post-arrest care (76%) [51]. While during CPR, the TEE protocol is usually simplified and limited to basic views after the ROSC extended protocol is executed, considering that patients remain usually unconscious or deeply sedated. In the specific setting of post-resuscitation care, it is desirable not to limit the evaluation to the heart and great vessels [24]. Indeed, the TEE approach allows transesophageal lung ultrasonography (TELUS) [52] and transgastric abdominal ultrasonography (TEGAUS) [53].

## 3. Post-Cardiac Arrest Syndrome: Pathophysiology and Echocardiographic Feature

Regardless of the primary cause, the survival of patients with OHCA is poor, estimated at less than 15%. Deaths occur due to post-cardiac arrest syndrome (PCAS), caused by the ischemia-reperfusion injury that affects the whole organism. This process is characterized by a sustained ischemic insult, which leads to cell damage; a shift to anaerobic metabolism with subsequent tissue acidosis; and an acid–base imbalance, which is responsible for several cellular dysfunctions. As a consequence of metabolic deviation, there is less ATP production and potentially an accumulation of toxic lipid substances, causing myocyte apoptosis, myocardial fibrosis, and, ultimately, cardiac dysfunction. In contrast, when reperfusion takes place, lipids once again become the main energy source, and oxygen consumption increases, leading to cardiac dysfunction as well [54].

Systemic acidemia resulting from hypoperfusion of vital tissues during CA is one of the predominant causes for its increased mortality, as it causes the impairment of cardiac function by reducing the perfusion of the myocardium. Acidosis can be both a cause and consequence of CA and is often associated with a poor prognosis and high mortality, which increases with the severity of the metabolic alteration. This condition determines a vicious circle, as lower CO with subsequent lower tissue perfusion increases the lactate blood level by worsening acidosis. Blood lactate concentrations may be considered as a marker of prolonged hypoperfusion or poor resuscitation, while lactate clearance during the first phase of post-resuscitation care may correlate with a more favorable outcome [55].

In the literature, few studies have examined the relationship between systemic acidosis and myocardial dysfunction, measured by invasive and non-invasive techniques. A fundamental role is played by TTE. Many data show that a reduced arterial pH is associated with impaired cardiac function and contractility [56]. Based on data derived from animal studies, lactic acidosis was associated with a notable reduction in SV and depressed left ventricle (LV) contractility potentially due to an increased end-diastolic volume resulting from acute pulmonary hypertension. In another similar study, respiratory acidosis showed decreased LV contractility and a fall in blood pressure due to a decreased SVR and impaired peripheral vascular reactivity to vasoactive drugs. These data support the hypothesis that a significant relationship between the fall of systemic pH and a reduction in cardiac function exists, although available data on human models is scarce, and no threshold for arterial pH has been established below which cardiac function is impaired [57].

Myocardial dysfunction occurring after successful resuscitation from CA is known as PAMD. It is attributed to myocardial stunning from global ischemia, regardless of the primary cardiac or non-cardiac causes. The manifestations of PAMD include global LV systolic dysfunction, LV diastolic dysfunction, or RV dysfunction. Among these, LV systolic impairment is the most common and significant manifestation, presenting as one of the following echocardiographic patterns: type I—global dysfunction; type II—RWMA; and type III—Takotsubo cardiomyopathy-like pattern (Takotsubo pattern) [58].

PAMD developed in the first 24 h post-ROSC, but it was a reversible process (meant as EF greater than 50% or disappearance of RWMA on follow-up echocardiography) and a return to normal global LV systolic function was registered by 72 h after ROSC.

The pathophysiological explanation of transient myocardial dysfunction after resuscitated CA is ascribed to ischemia-reperfusion injury and the inflammatory response after ROSC, associated with high levels of plasma catecholamines. This latter mechanism could be responsible for both the Takotsubo pattern (as in stress-induced cardiomyopathy) and RWMA (in patients with underlying unrevealed CAD) [28,58].

Human studies suggest that about two-thirds of patients resuscitated from CA present LV systolic dysfunction within the first 24 h after ROSC, with a mean LV ejection fraction (LVEF) between 35 and 45% [59]. A persistently low cardiac index (CI) at 24 h was associated with early death, but in the surviving patients, normal hemodynamics was restored by 72 h. Apical segments displayed more severe RWMA with the sparing of basal segments, a finding also seen in stress cardiomyopathy. PAMD shares characteristics with stress-induced or septic cardiomyopathy [54], which is not necessarily related to coronary stenosis.

Often, echocardiography is the first-line diagnostic tool for the individuation of PAMD, of which reduced LVEF is the most reported manifestation. It is still difficult to determine whether myocardial dysfunction encountered at TTE echocardiography is related to PCAS or is the red flag of significative coronary stenosis [60].

### 3.1. Role of Echocardiography in Post-Arrest Management of Suspected ACS/CAD

The early revascularization of obstructed coronary arteries can reduce myocardial damage and its consequences, such as ventricular dysfunction, rhythm disturbances, heart failure, and death [54].

Although coronary angiography after CA is of potential diagnostic and therapeutic relevance when acute coronary occlusion is present, the timing of coronary angiography in patients without ST-segment elevation remains one of the most controversial decisions to make in post-CA.

The difficulty encountered in this scenario is that, even in the case of coronary cause, it is not so easy to distinguish between acute coronary events and pre-existing CAD [54].

The role of echocardiography in this setting is limited. A considerable amount of myocardial dysfunction detected through TTE in post-CA could be attributed to a pre-existing or new cardiac injury, especially those presenting with new RWMA, which could be related to underlying coronary stenosis in need for urgent angiography [55]. Nonetheless, as mentioned before, such findings are not specific as they might be manifestations of PAMD, so echocardiography should be considered as an adjunctive piece in guiding global management of the patient, but it does not provide information that can help clinicians in the determination of the timing of coronary angiography.

However, two randomized prospective trials, the TOMAHAWK trial (Angiography after Out-of-Hospital Cardiac Arrest without ST-Segment Elevation) and COACT trial (Coronary Angiography after Cardiac Arrest without ST-Segment Elevation), have already shown that a strategy of immediate angiography was not found to be better than delayed angiography in terms of prognostic meaning [61,62].

### 3.2. Role of Non-Cardiac Ultrasounds

Beyond the role of echocardiography, non-cardiac US also assumes a key position in post-RESC management. Most resuscitated patients require mechanical ventilatory support. LUS is a practical bedside tool that could provide relevant information in the immediate post-ROSC phase and could be easily used for the daily monitoring of the patient. LUS demonstrating bilateral ventilation contributes to the confirmation of the correct placement of the endotracheal tube during or immediately after RCP. It also enables the identification of pulmonary disease that could cause hypoxia as a reversible cause of CA or pulmonary injuries that could represent a complication of RCP. Particularly, pneumothorax, pleural effusion, and consolidation, both caused by atelectasis and pneumonia, may be easily detected [22]. As already mentioned, abdominal US could be used to detect massive intrabdominal bleeding and organ injury. Moreover, the examination of intra-abdominal organ hemodynamics using Doppler studies contributes to the evaluation of organ perfusion [43].

LUS can accurately diagnose CA by drowning. LUS showed multiple B-lines on the anterior and lateral surfaces of both lungs, consistent with pulmonary edema. Transthoracic echocardiography assessed with focus showed no pericardial effusion and normal global left ventricular function. In conclusion, it is non-cardiogenic pulmonary edema [63].

Post-resuscitation cerebral hemodynamics can be assessed by transcranial Doppler (TCD) by examining waveform patterns, the pulsatility index, and the mean flow velocity in the major cerebral arteries. Continual TCD assessments and optic nerve sheath diameter evaluations via the US can aid in non-invasively evaluating cerebral edema resulting from ischemia-reperfusion syndrome and could help in the determination of brain stem death, particularly in the case of post-traumatic CA [8,64]. Finally, the US, beyond the role in facilitating venous and arterial cannulation, allows the monitoring of vascular complications associated with invasive monitoring and support [8].

## 4. Prognostic Role of Echocardiography in Resuscitated CA Patients: A Future Perspective

As already mentioned before, PAMD generally recovers by 72 h after resuscitation. On the contrary, if PAMD is persistent after 72 h, it is often associated with a worse prognosis [55]. Interestingly, a scoping review by Liu et al. [28], analyzing data already present in the literature, showed that post-arrest LVEF is not associated with statistical changes in survival or neurologic outcomes [35,65]. On the contrary, two different studies demonstrated that echocardiographic parameters with prognostic meaning associated with worse survival to hospital discharge, independent of LV systolic function, were reduced. Right ventricular global systolic function (measured by RV fractional area change and 3D RV ejection fraction) [66] and the diastolic parameters of the ratio of early mitral Doppler filling and mitral annular excursion (E/e’) were representative of LV diastolic function and filling pressures [67]. Particularly, one study has suggested that LV diastolic dysfunction, rather than LV systolic dysfunction, is associated with increased mortality after OHCA [68].

Jentzer et al. emphasize the fundamental utilization of echocardiography not just for the early, single evaluation of post-ROSC cardiac function but in the assessment of LVEF changes through serial TTE. The results show that no significative difference in the initial LVEF between long-term survivors and non-survivors was found, while survivors had greater increases in the markers of systolic function than non-survivors, highlighting that dynamic changes in systolic function are associated with outcomes after OHCA more than single static measurements [35]. These data keep in line with the previously discussed concept that some of the observed changes in TTE parameters after CA could be due to transient myocardial stunning and are therefore reversible. Prognostic TEE parameters are summarized in Table 6.

## 5. Conclusions

The use of focused TTE and TEE during the resuscitation of patients in CA and peri-arrest states has shown to be feasible and clinically impactful in peri-operative, intensive care, and emergency settings. By synthesizing the existing literature, we seek to identify specific echocardiographic patterns that can be utilized to stratify patients both diagnostically and prognostically. Understanding these patterns may enhance the ability to predict outcomes and tailor post-arrest interventions more effectively, ultimately improving survival and functional recovery in this vulnerable patient population. Future research should include larger studies evaluating the diagnostic value and hemodynamic and clinical impact of echocardiography-guided resuscitation.

## Figures and Tables

**Table 1:** Goals of ultrasounds during cardiopulmonary resuscitation and after restoration of spontaneous circulation.

Goals of the US During CPR	Goals of the US in Post-Resuscitation Care
Diagnosis of reversible causes	Diagnosis of the underlying cause of CA
Confirm the effectiveness of chest compressions	Hemodynamic monitoring and optimization
Determine the presence of cardiac contractions or “standstill”	Assist ventilatory support
Confirm bilateral ventilation after intubation	Assessment of CPR complication
Assist invasive procedures (pericardiocentesis, vascular cannulation, extracorporeal CPR)	Assessment of multiorgan function (prognosis)
	Assist invasive procedures

CA: cardiac arrest; CPR: cardiopulmonary resuscitation; US: ultrasound.

**Table 2:** Ultrasound signs for reversible causes.

Potential Cause	US Views	Suggestive Findings	Intervention
**Profound hypovolemia**	Subcostal Abdomen	Small LV and RV cavity size Near end-systolic obliteration (“kissing ventricle”) Collapsed IVC (<10 mm) Massive bleeding in the abdomen	Fluid administration; assess response
**Cardiac** **tamponade**	Subcostal	Pericardial effusion Collapsed cardiac chambers Congested IVC	Pericardiocentesis; guide the procedure and assess the response
**Massive pulmonary embolism**	Subcostal Lower limbs	Markedly dilated RV Pressure overload of RV Thrombus-in-transit Congested IVC Presence of DVT (positive CUS)	Consideration of thrombolysis
**Tension** **pneumothorax**	Lung	Absence of lung sliding during ventilation	Needle decompression, assess response

CUS: compression ultrasound; DVT: deep vein thrombosis; IVC: inferior cava vein; LV: left ventricle; RV: right ventricle; US: ultrasounds.

**Table 3:** Transesophageal echocardiography during cardiopulmonary resuscitation.

View	Goals and Diagnosis
**1.** **ME 4C (0–10°)**	Tamponade Evaluation of LV/RV contractility Signs of PE Signs of profound hypovolemia Signs of compression due to pneumothorax
**2.** **ME LAX (120–140°)**	Determine AMC Optimization of chest compression avoiding LVOT obstruction Evaluation of AscAo
**3.** **TG SAX (0–20°)**	Tamponade Evaluation of LV/RV contractility Signs of PE Signs of profound hypovolemia;
**4.** **ME bicaval (90°)**	Evaluation of intravascular volume (SCV) Thrombus in transit Assist venous procedures
**5.** **TG and ME DescAO SAX (0–10°)**	Evaluation of DescAo Assist arterial procedures

4C: 4-chamber; AMC: area of maximal compression; AscAo: ascending aorta; DescAo, descending aorta; LAX, long axis; LVOT: left ventricular outflow tract; ME: midesophageal; PE: pulmonary embolism; SAX, short axis; SCV: superior cava vein; TG: transgastric; LV/RV: left ventricle/right ventricle.

**Table 4:** Main echocardiographic features of acute pulmonary embolism.

Echocardiographic Findings	Parameters
**RV dilatation**	RV/LV ratio > 1 RV basal diameter > 41 mm RV mid diameter > 35 mm
**RV systolic disfunction**	TAPSE < 17 mm S’ wave (TDI) < 10 cm/s RV-FAC < 35% RV Tei index (PW) > 0.43 RV Tei index (TDI) > 0.54 RV free wall strain > −20%
**McConnell Sign**	RV basal and mid-free wall akinesia and normal motion of the RV apex
**RV pressure overload**	TR Vmax > 2.9 m/s Pulmonary flow AcT < 60 msec Pulmonary flow mid-systolic notch Paradoxical IVS motion Flattened IVS with D-shaped LV Dilated PA (>25 mm) TAPSE: PASP ratio < 0.4 Dilated IVC (>21 mm) and/or diminished collapsibility
**60/60 Sign**	TR jet gradient < 60 mmHg and Pulmonary AcT < 60 ms
**Thrombus in transit**	Thrombus in RV, RA or PA

AcT: acceleration time; FAC: fractional area change; IVS: interventricular septum; LV: left ventricle; PA: Pulmonary artery; PASP: pulmonary artery systolic pressure; PW: pulsed wave; RA: right atrium; RV: right ventricle; TAPSE: tricuspid annular planar systolic excursion; TDI: tissue Doppler imaging; TR: tricuspid regurgitation.

**Table 5:** Ultrasound parameters for hemodynamic monitoring and optimization.

	Parameter	Utility	How to Calculate	Normal Values and Interpretation
**Perfusion** **parameters**	**LVOT VTI**	The distance that blood travels across the LVOT during the cardiac cycle	Tracing the PWD spectral display of the LVOT	LVOT-VTI > 18 cm
	**SV**	The volume of blood pumped during each systolic cardiac contraction	SV = LVOT area * x LVOT-VTI SVi= SV/BSA	SV > 70 mL SVi >35 mL/mq
	**CO and CI**	Amount of blood pumped by the heart in a minute;	CO = SV × HR CI = CO/BSA	CO > 4 l/min CI < 2.5 l/min/mq
**Preload parameters (fluid responsiveness and fluid tolerance)**	**IVC diameter and collapsibility**	Used to estimate RA pressure and volemic status	Diameters of IVC at end-expiration and inspiration in subcostal view	IVC < 21 mm that collapses > 50% (RAP 0–5 mmHg); IVC > 21 mm that collapses > 50% or IVC < 21 mm that collapses < 50% (RAP 5–10 mmHg); IVC > 21 mm that collapses < 50% (RAP 10–20 mmHg)
	**JVD ratio**	Used to estimate RA pressure and volemic status	JVD during Valsalva/JVD at rest	JVD ratio < 3 suggests elevated RAP and fluid overload
	**LVOT-VTI variability**	Dynamic parameters that suggest fluid responsiveness	Evaluation of LVOT-VTI in different respiratory phases during MV, after PLR or fluid challenge	Change in LVOT-VTI < 10–15% indicates fluid responsiveness
	**VExUS score**	Evaluation of systemic congestion in four grades	Combined evaluation of IVC diameter and venous flow pattern using PWD in HV, PV, and IRV	VExUS score 0 = no congestion; VExUS score 3 = severe congestion
	**LUS B-lines**	Evaluation of pulmonary congestion	Evaluation of B-lines in 8 to 12 zones	B-lines < 3 for scanning zone = normal; Multiple and diffuse B-lines = severe congestion
	**E/e’**	Marker of LV filling pressure that correlates with PCWP [ PCWP ≈ 1.24 × (E/e) + 1.9]	The ratio between mitral inflow E velocity using PWD and e’ lateral and medial velocity using TDI	E/e’ < 7 = normal filling pressure; E/e’ > 15 = elevated filling pressure
**Afterload** **parameters**	**SVR**	Determinant of LV afterload and reflects the tone of systemic blood vessels.	MAP-CVP/CO **	SVR 800–1200 dynes·s/cm^5^= 10–15 WU
	**PASS**	Estimation of pulmonary artery systolic pressure	PASP = 4 × (TRV^2^) + RAP	PASP < 35 mmHg
	**PAMP**	Estimation of pulmonary artery mean pressure	PAPM = 0.61 × PASP + 2 or PAPM = 4 × (PRV^2^) + RAP	PAMP < 20 mmHg
	**PVR**	Determinant of RV afterload and reflect the tone of pulmonary blood vessels	PVR = (PAMP-PCWP)/CO	PVR < 2 WU
	**TRV/RVOT-VTI ratio**	Parameter to estimate PVR and PAP	The ratio between TRV and RVOT-VTI was calculated tracing the PWD spectral display of the RVOT.	TRV/RVOT-VTI ratio < 0.45

BSA: Body Surface Area; CI: Cardiac Index; CO: Cardiac Output; CVP: Central Venous Pressure; HV: Hepatic Vein; IVC: Inferior Vena Cava; JV: Jugular Vein; LV: Left Ventricle; LVOT: Left Ventricular Outflow Tract; LUS: Lung Ultrasound; MAP: Mean Arterial Pressure; MV: Mechanical Ventilation; PAMP: Pulmonary Artery Mean Pressure; PASP: Pulmonary Artery Systolic Pressure; PCWP: Pulmonary Capillary Wedge Pressure; PLR: Passive Leg Raise; PRV: Pulmonary Regurgitation early diastolic Velocity; PVR: Pulmonary Vascular Resistance; PV: Portal Vein; PWD: Pulsed-Wave Doppler; RA: Right Atrium; RAP: Right Atrial Pressure; RV: Right Ventricle; RVOT: Right Ventricular Outflow Tract; SV: Stroke Volume; SVR: Systemic Vascular Resistance; SVi: Stroke Volume Index; TDI: Tissue Doppler Imaging; TRV: Tricuspid Regurgitation Velocity; VExUS: Venous Excess Ultrasound Score; VTI: Velocity Time Integral; WU: Wood Units. * LVOT area is obtained by measuring the LVOT diameter in the parasternal long-axis (PLAX) view during mid-systole. ** MAP is measured invasively or non-invasively; RAP could be assessed using a central vein catheter (CVC) or estimated through the US evaluation of IVC diameter and collapsibility; CO is calculated using the LVOT area and VTI.

**Table 6:** Prognostic TTE parameters.

	Parameters	THE	Notes
**Systolic function**	Serial LVEF assessment	LVEF evaluated through the Biplane method	Dynamic changes in systolic function are associated with outcomes after OHCA more than single static measurements.
	RV function	RV FAC and 3D RV ejection fraction *	Reduced RV systolic function (RV FAC < 35% or 3D RV ejection fraction < 45%) associated with worse outcome
**Diastolic function**	LV diastolic function and filling pressures	Ratio of early mitral Doppler filling and mitral annular excursion (E/e’) *	LV diastolic dysfunction (E/e’ > 14) is associated with increased mortality after OHCA.

FAC: fractional area change; LV: left ventricle; LVEF: left ventricle ejection fraction; OHCA: out-of-hospital cardiac arrest; RV: right ventricle; TTE: transthoracic echocardiography. * Independently of LV systolic function.

## Data Availability

No new data were created or analyzed in this study. Data sharing is not applicable to this article.
